# Formability and Failure Mechanisms of Woven CF/PEEK Composite Sheet in Solid-State Thermoforming

**DOI:** 10.3390/polym11060966

**Published:** 2019-06-03

**Authors:** Bing Zheng, Xiping Gao, Maoyuan Li, Tianzhengxiong Deng, Zhigao Huang, Huamin Zhou, Dequn Li

**Affiliations:** State Key Laboratory of Material Processing and Die & Mold Technology, Huazhong University of Science and Technology, Wuhan 430074, China; zhengbing@hust.edu.cn (B.Z.); gaoxiping@hust.edu.cn (X.G.); limaoyuan@hust.edu.cn (M.L.); uezuuezu@hust.edu.cn (T.D.); hmzhou@hust.edu.cn (H.Z.); ldq@hust.edu.cn (D.L.)

**Keywords:** woven CF/PEEK, solid-state thermoforming, formability, flexural test, Erichsen test, failure mechanisms

## Abstract

In this study, the formability of woven carbon-fiber (CF)-reinforced polyether-ether-ketone (PEEK) composite sheets in the solid-state thermoforming process were investigated, and the failure mechanisms were discussed. The formability of the woven CF/PEEK sheets were analyzed using flexural tests, Erichsen test, and microscopic observation. The results show that the formability of CF/PEEK sheets significantly increases as the temperature rises from 165 to 325 °C, and slightly decreases as the deformation speed rises from 2 to 120 mm/min. The deformation of the sheets is caused by plastic deformation, shear deformation and squeeze deformation, without plastic thinning and fiber slippage, which is due to the restriction of the solid matrix and locked fibers. Moreover, the wrinkles will cause fiber fracture at lower temperatures and delamination at higher temperatures. At higher temperatures, the wrinkles mainly occur at the position with [0°/90°] fibers due to the squeezing of the matrix and fibers.

## 1. Introduction

In the last three decades carbon fiber (CF)-reinforced polymer composites (CFRP) have made their way into the aerospace and automotive industries as structural elements because of their high specific stiffness and strength [1,2,3]. Recently, there has been increased interest in CF-reinforced thermoplastic (CRTP) composites, especially high-performance polyether-ether-ketone (PEEK) composites, because of their numerous advantages, such as excellent strength and stiffness, good toughness, and high service temperatures, which make them widely used in fields such as aerospace, mechanical, electrical, and biological engineering [4,5,6,7].

A pre-prepared sheet is the common form for CF/PEEK composites [8,9,10], and it is then formed into the required part by solid-state thermoforming. In previous work, the woven CF/PEEK sheets were prepared, and the process-structure-properties relationship was systematically discussed [11]. In order to thermoform the prepared CF/PEEK sheets into the required part, it is necessary to study the formability of CF/PEEK sheets in the solid-state thermoforming process. Solid-state thermoforming [12,13] is a manufacturing method by heating CFRTP sheets to a pliable state, then pressing the sheets against a cool mold, holding the formed sheet against the mold until cool. Compared with the traditional manufacturing methods, include compression molding, extrusion forming and tape winding [14,15,16], the thermoforming of solidified CFRTP sheets can reduce the production time and may increase the flexibility of the manufacturing process. Since the semi-crystalline thermoplastic matrix can be permanently deformed between the glass transition temperature (T_g_) and the melting point (Tm) [17], it does not have to be cooled from the melt state to a solid; the cycle times for this process are very short, typically less than fifteen seconds. Thence, the solid-state thermoforming of CFRTP composites has become a popular research area in recent decades.

In the 1980s, Bigg et al. [18] investigated the solid-state stamping process of glass-fiber-reinforced thermoplastic (GFRTP) sheets. They found that solid-state stamping is a viable option for producing simple parts of semi-crystalline thermoplastic matrix composites sheet, and the feasible temperature region was found to be 25–30 °C below the T_m_ of the polymer. Later, Cabrera et al. [19] compared the solid-state thermoforming behavior of self-reinforced PP and GF/PP composite sheets. Compared with GF/PP composites, self-reinforced PP composites can be fabricated by solid-state thermoforming to produce geometrically complex parts, and the mode of deformation are shear deformation and fiber deformation. Afterward, Jun Yanagimoto et al. [20] proposed a new sheet forming process for solidified CFRP at room temperature and at 200 °C, in which a UD-CFRTS sheet was sandwiched by dummy metallic sheets during stamping. It was found that the proposed forming method combined with the high-speed manufacturing of thermosetting composite sheets will markedly improve the productivity of parts. In particular, Yu et al. [21,22,23,24] systematically studied the cold and warm stamping formability of UD-CF/EP sheets by V-bending, deep drawing, and the Erichsen hemispherical stamping test. The results show that there is a certain relationship between the forming conditions and the basic properties, such as tensile strength and structure of the product, and the failure of the composite sheets are mainly due to fiber disarray and delamination. Moreover, the UD-CF/EP composite sheet can be stretching at 100 °C.

However, the literature about the formability of woven CF/PEEK sheet during solid-state thermoforming has not been observed. Therefore, the purpose of this paper can be divided into three purposes: (1) determine the feasibility for the solid-state thermoforming of woven CF/PEEK sheets; (2) obtain the corresponding formability and optimal forming parameters; and (3) discuss the failure mechanism during the solid-state thermoforming. There are four main forming tests to investigate the formability of metal sheets: bending, deep drawing, stretching, and stretch flanging. Although these tests are conducted for metals, some of the knowledge can be applied to CFRP sheets, and the mechanism of bending and stretching deformation is a concerned topic in the forming of CFRP [21,24,25].

In this study, the formability of woven CF/PEEK sheet in the solid-state thermoforming process were investigated. First, the T_g_ of PEEK and CF/PEEK was measured by dynamic mechanical analysis (DMA) tests. Then, the formability of woven CF/PEEK sheets at elevated temperatures (T_g_ < T < T_m_) were analyzed using flexural tests and the Erichsen test. Furthermore, the microstructure was observed using an optical microscope and a scanning electron microscope (SEM). Finally, the failure mechanism of the flexural tests and Erichsen test were systematically discussed.

## 2. Materials and Methods

### 2.1. Materials

**Figure 1:**
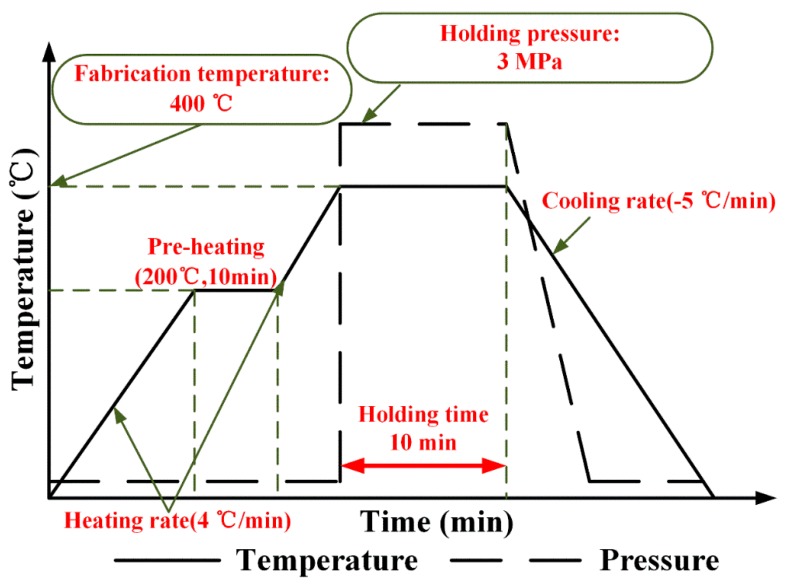
Compression molding process of woven CF/PEEK composite sheets.

Based on the previous work [11], five woven CF/PEEK pre-pregs were stacked along the [0/90] direction in the mold, and fabricated into the composite sheets by compression molding. The fabricated process was 400 °C/3 MPa/10 min, and shown in Figure 1. The fiber mass fraction of woven composite sheets was 69 ± 1%. The sheets consisted of plain-weave CF fabrics (T300 3K, supplied by the TORAY Company, Tokyo, Japan) and PEEK resin (Grade 150P, supplied by the Victrex Company). The test specimens were sawn from plates, with dimensions of 140 mm × 140 mm, using a computer numerical control machine and the thickness of the specimens was 0.9 ± 0.1 mm. They were subsequently dried at 60 °C for 1.5 h.

### 2.2. DMA Test

DMA tests were performed in a dynamic thermomechanical analysis machine (Diamond DMA, PerkinElmer Instruments, Shanghai, China) to investigate the T_g_ of PEEK and CF/PEEK. Rectangular specimens with approximate dimensions of 40 mm × 5 mm × 0.9 mm were tested in three-point flexural mode, and the span was 20 mm. The strain history was a sinusoid with a peak amplitude of 0.01%, and the temperature was swept from 20–235 °C at a heating rate of 3 °C/min with frequencies of 1 Hz. The material was subjected to cyclic stress during the DMA test, and the viscoelastic properties of the material could be characterized by the stress response and strain response recorded in the experimental process. The relationship between storage modulus (E′), loss modulus (E″), loss tangent (tan δ), and temperature can be directly measured from the DMA test. The corresponding loss tangent (tan δ) was measured to confirm the T_g_ of PEEK and CF/PEEK.

### 2.3. Flexural Tests

**Figure 2:**
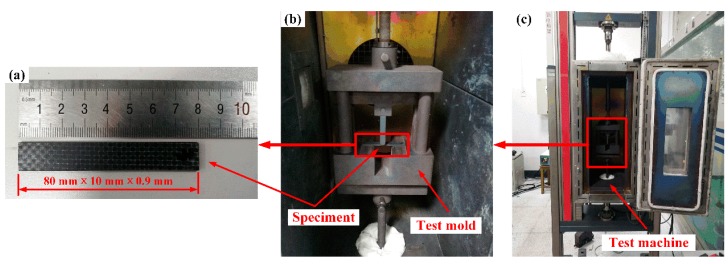
Representation of Flexural test at elevated temperatures: (**a**) Specimens; (**b**) test mold; and (**c**) test equipment.

**Figure 3:**
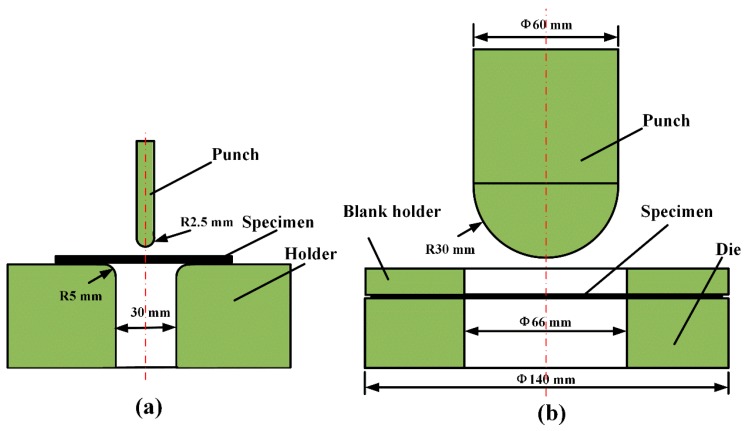
Schematic diagram of flexural and Erichsen tests at elevated temperature: (**a**) Flexural test; and (**b**) Erichsen test.

Flexural tests were performed using an electrical universal testing machine (Z020, Zwick/Roell, Ulm, Germany). Rectangular specimens with approximate dimensions of 80 mm × 10 mm × 0.9 mm were tested, and the span was 40 mm. The flexural behavior and formability of woven CF/PEEK sheets were investigated over a wide range of temperatures (165–325 °C) and deformation speeds (2–200 mm/min). The specimens were kept at room temperature for three days before testing, and three samples were tested for each experimental condition. Figure 2 and Figure 3a show the specimens, mold, and test equipment.

The flexural strength, *σ_f_*, modulus, *E_f_*, and failure angle Θ were calculated using Equations (1)–(3):

(1) $$\sigma_{f}=\frac{3Pl_{f}}{2b_{f}h_{f}^{2}}$$

(2) $$E_{f}=\frac{l_{f}^{3}\Delta P}{4b_{f}h_{f}^{3}\Delta S}$$

(3) $$\Theta =\arctan(\frac{l_{f}}{S})$$

where failure angle Θ is the parameter defined by the author to characterize the flexural ability of the material. *P* is the maximum load at the point of first failure, and *S* is the deflection; *b_f_*, *h_f_*, and *l_f_* are the width, thickness, and span of the specimens, respectively; and ∆*P* and ∆*S* are the load increment and deflection increment of the linear portion of the load-deflection curve, respectively.

### 2.4. Erichsen Tests

**Figure 4:**
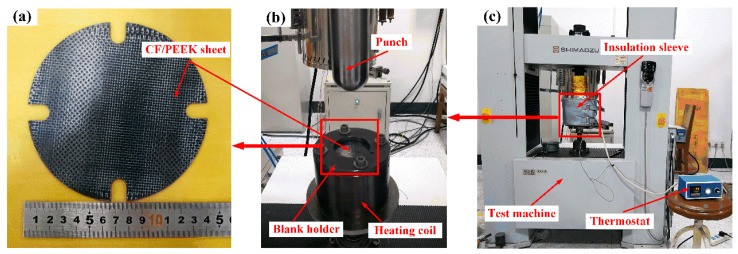
Representation of the Erichsen test at elevated temperatures: (**a**) Specimens; (**b**) test mold; and (**c**) test equipment.

The Erichsen test is one of the popular formability tests to simulate the stretching deformation of sheets [24,26,27], which were selects to investigate the stretching formability of woven CF/PEEK sheet in the solid-state thermoforming. Erichsen test were performed using a material high temperature durability test machine (AG-IC 100Kn, Shimadzu, Kyoto, Japan). Round specimens with approximate dimensions of ϕ 138 mm × 0.9 mm were tested. The Erichsen formability of woven CF/PEEK sheets were investigated over a wide range of temperatures and deformation speeds. Table 1 show the Ericksen test conditions, and Figure 3b and Figure 4 show the specimens, mold, and test equipment.

In the Ericksen test, the mold and blank holder were heated by the heating coil, which heats the CF/PEEK sheet to the specified temperature by superheat conduction. The spherical punch was pressed into the sheet until cracks occur, and the experimental data of load (F) and displacement (H) were saved. The index of Ericksen (*IE*) and limit Ericksen ratio (*LER*) were calculated using Equations (4) and (5):

(4) $$IE=H_{f}$$

(5) $$LER=\frac{IE}{D_{Er}}$$

where, *H_f_* was the displacement of punch when the sample was broken, and *D_Er_* was the diameter of the cup punch.

### 2.5. Microstructure Observation

The microstructures of the failure specimens were observed using a super depth-of-field three-dimensional digital microscope (VHX-1000C, KEYENCE, Osaka, Japan) and a field emission scanning electron microscope (JSM-7600F, JEOL, Beijing, China). The optical microscope samples were polished to expose a fractured cross-section for observation. The SEM samples were sprayed with gold for 300 s to enhance the conductivity of the fracture surface before the test. The fractography analysis of the failed specimens were proved to be sufficiently relevant to study the failure mechanisms [28].

## 3. Results and Discussions

### 3.1. DMA Analysis

**Figure 5:**
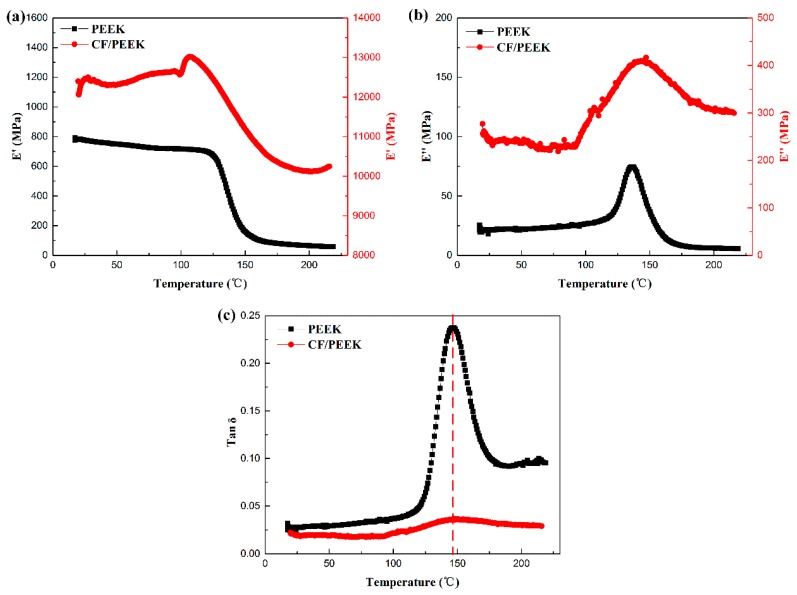
DMA results of PEEK and woven CF/PEEK composites: (**a**) the storage modulus (E′) versus temperature; (**b**) the loss modulus (E″) versus temperature, and (**c**) the tan δ versus temperature.

Figure 5 is the DMA results of PEEK and woven CF/PEEK composites. The E′, E″, and tan δ versus temperature curves are presented in Figure 5a–c, respectively. When the polymer begins to undergo a glass transition, the tan δ value will reach the maximum [29], which means the T_g_ of PEEK and woven CF/PEEK are the same, both of them are 148 °C. Simultaneously, according to the previous work [11], the Tm of the woven CF/PEEK sheets is 340 °C. In order to ensure that the matrix of the sheets is in a rubbery state, the experimental temperature is chosen from 165–325 °C.

### 3.2. Flexural Properties and Formability at Various Temperatures

**Figure 6:**
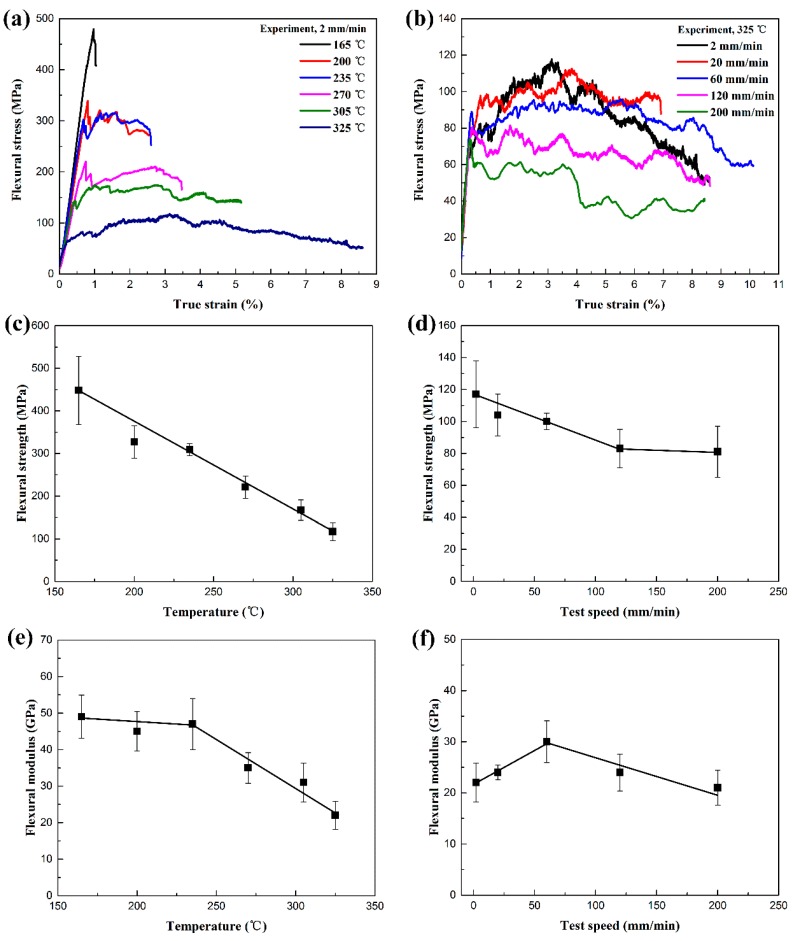
Flexural properties of woven CF/PEEK composites at different condition: (**a**,**b**) flexural stress-strain curve, (**c**,**d**) the relationship between flexural strength and temperature or speed, and (**e**,**f**) the relationship between flexural modulus and temperature or speed.

Figure 6 shows the flexural properties of woven CF/PEEK composites at different condition. As shown in Figure 6a, the woven CF/PEEK composite exhibits a typical brittle fracture behavior when the deformation temperature is 165 °C. When the deformation temperature is in the range of 200–325 °C, the CF/PEEK composite exhibits viscoelasticity deformation behavior, which includes an elastic phase and visco-plastic phase. As the deformation temperature increases, the elastic phase of the curve decreases slightly, and the visco-plastic phase increases substantially. As shown in Figure 6b, when the deformation speed is in the range of 2–60 mm/min, the deformation of CF/PEEK composite is mainly divided into an elastic phase, yield phase, and post-yielding phase showing a strain hardening behavior and strain softening phenomenon. As the deformation speed increases from 120 to 200 mm/min, the curve does not exhibit strain hardening behavior. As shown in Figure 6c,e, the flexural strength of woven CF/PEEK composite decreases approximately linearly, decreasing from 447 MPa to 117 MPa as the deformation temperature increases from 165 to 325 °C; the flexural modulus slightly decreases from 49 GPa to 47 GPa as the deformation temperature increases from 165 to 235 °C, and then significantly reduce to 22 GPa when the deformation temperature is 325 °C. As shown in Figure 6d,f, the flexural strength of CF/PEEK composite decreases from 117 MPa to 83 MPa as the speed increases from 2 to 120 mm/min. It then slightly decreases to 81 MPa when the speed is 200 mm/min. The flexural modulus of CF/PEEK composite tends to increase from 22 GPa to 30 GPa as the speed increases from 2 to 60 mm/min, and then decreases to 21 GPa when the speed is 200 mm/min.

**Figure 7:**
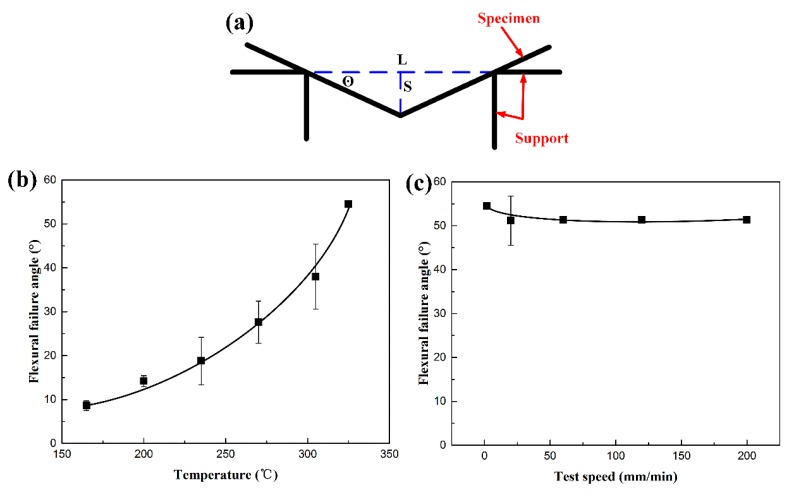
Relationship between flexural failure angle and deformation condition of woven CF/PEEK composites: (**a**) schematic diagram of flexural failure angle, (**b**) the relationship between failure angle and deformation temperature, and (**c**) the relationship between failure angle and deformation speed.

Figure 7 shows the relationship between the flexural failure angle and deformation condition of woven CF/PEEK composites. As shown in Figure 7a, the flexural failure angle Θ refers to the angle which the material is bent when the flexural fails. Larger values of Θ indicates better flexural deformability. As shown in Figure 7b, the flexural failure angle of woven CF/PEEK composites increases gradually from 8.6° to 54.5° as the deformation temperature increases from 165 to 325 °C, representing that the flexural deformability of CF/PEEK composites sheets gradually increases with the increase of temperature. As shown in Figure 7c, the deformation speed has little effect on the flexural deformability of CF/PEEK at 325 °C. When the deformation speed increases from 2 to 200 mm/min, the flexural failure angle drops slightly, but all of which are greater than 50°.

**Figure 8:**
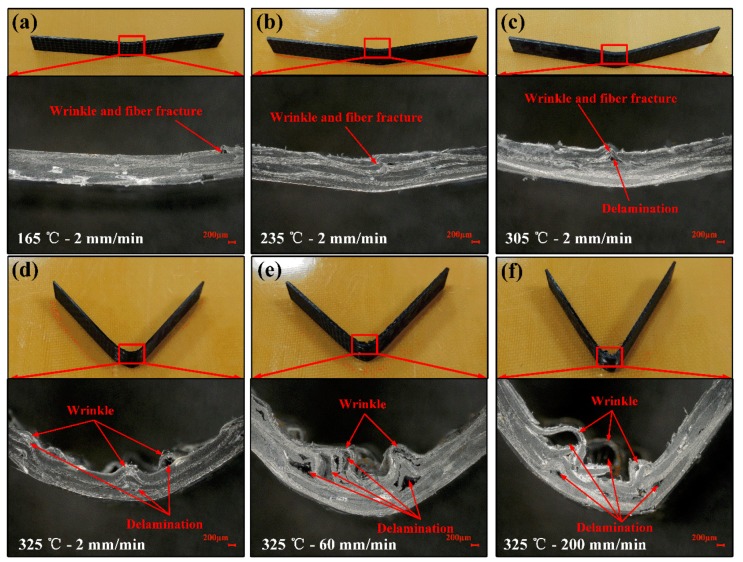
Micrographs of the fracture surfaces of woven CF/PEEK composites after flexural testing under different conditions: (**a**) 165 °C—2 mm/min, (**b**) 235 °C—2 mm/min, (**c**) 305 °C—2 mm/min, (**d**) 325 °C—2 mm/min, (**e**) 325 °C—60 mm/min, and (**f**) 325 °C—200 mm/min.

Figure 8 shows the micrographs of the fracture surfaces of woven CF/PEEK composites after flexural testing under different conditions. As shown in Figure 8a,b, the specimens are less deformed, and the fractures exhibit small wrinkles and fiber breakage in the temperature range of 165–235 °C. As the temperature rises to 305 °C, the deformation of the sample is increased, and the fracture exhibits medium-sized wrinkles, fiber breaks, and interlayer delamination, as shown in Figure 8c. As shown in Figure 8d, the sample is deformed greatly, and the fracture mainly exhibits large wrinkles and interlayer delamination as the deformation temperature is raised to 325 °C. As shown in Figure 8e,f, the deformation of the specimen is still large, and the fractures show more wrinkles and interlayer delamination when the deformation speed is increased to 60 and 200 mm/min.

**Figure 9:**
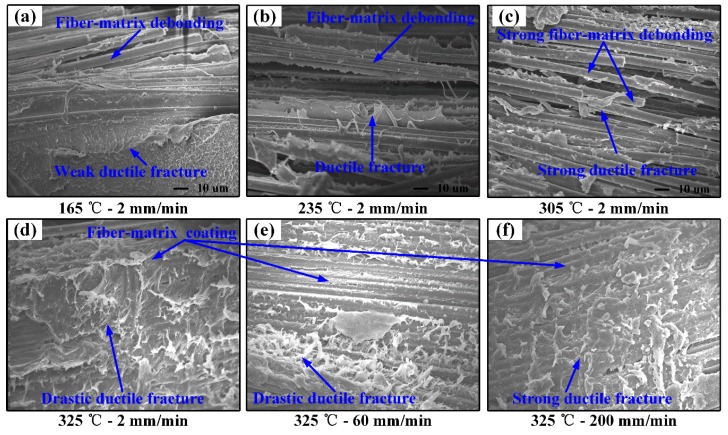
SEM images of the fracture surfaces of woven CF/PEEK composites after flexural testing under different conditions: (**a**) 165 °C—2 mm/min, (**b**) 235 °C—2 mm/min, (**c**) 305 °C—2 mm/min, (**d**) 325 °C—2 mm/min, (**e**) 325 °C—60 mm/min, and (**f**) 325 °C—200 mm/min.

Figure 9 shows the SEM images of the fracture surfaces of woven CF/PEEK composites after flexural testing under different conditions. The microscopic failure of FRP composites is generally due to matrix fracture and fiber/matrix debonding [30,31]. As shown in Figure 9a, the fracture exhibits the weak ductile fracture of the matrix and a small amount of fiber/matrix debonding when the temperature is 165 °C, which indicates relatively weak toughness and strong interfacial force. When the temperature rises to 235 °C, the toughness of the matrix increases and the interaction of fiber/matrix decreases, the fracture exhibits ductile fracture of the matrix and more fiber/matrix debonding, as shown in Figure 9b. When the temperature rises to 305 °C, the toughness of the matrix is extremely strong and the fiber/matrix interaction is greatly reduced. The fracture exhibits strong toughness fracture of the matrix and a large amount of fiber/resin debonding, as shown in Figure 9c. When the temperature rises to 325 °C, the fracture exhibits drastic ductile fracture of matrix, as shown in Figure 9d. As shown in Figure 9e, when the deformation speed is 60 mm/min, the increase in speed has no obvious effect on the toughness of the matrix, and the fracture still exhibits a drastic ductile fracture of the matrix. When the deformation speed rises to 200 mm/min, the toughness of the matrix is slightly weakened, and the fracture shows a strong toughness fracture of the matrix, as shown in Figure 9f. Moreover, in the speed range of 2–200 mm/min, the fibers are coated well by the matrix, and no firer/matrix debonding occurs. This means that the softening effect of temperature on the matrix is greater than the strengthening effect of the deformation speed on the matrix when the deformation temperature is 325 °C, and that the matrix first breaks due to lower strength.

### 3.3. Erichsen Formability at Various Temperatures

**Figure 10:**
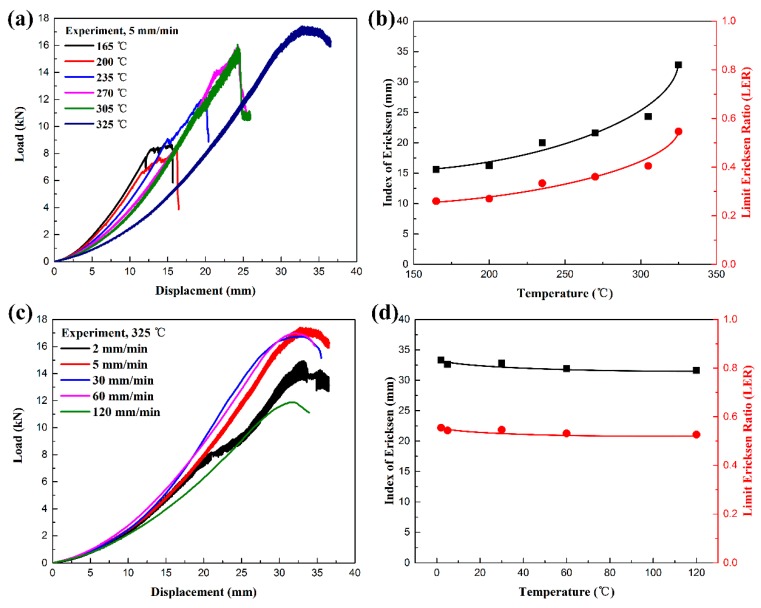
Erichsen forming performance of woven CF/PEEK sheets under different conditions: (**a**) force vs. displacement curve at different temperatures, (**b**) the relationship between IE and LER vs. temperature, (**c**) force vs. displacement curve at different test speed, and (**d**) the relationship between IE and LDR vs. test speed.

Figure 10 shows the Erichsen forming performance of woven CF/PEEK sheets under different conditions. As shown in Figure 10a,b, the Erichsen formability of woven CF/PEEK sheets gradually increases as the deformation temperature is increased from 165 to 325 °C. The IE value and LER rise from 15.6 mm and 0.26 to 32.8 mm and 0.55, respectively. Especially, when the deformation temperature is 325 °C, the IE and LER increase greatly, and the sheets exhibit strong formability. As shown in Figure 10c,d, the Erichsen formability of woven CF/PEEK composite sheets slightly decrease as the deformation speed is increased from 2 to 120 mm/min. The IE value and LER slightly reduce from 33.3 mm and 0.56 to 31.6 mm and 0.53, respectively. Comparing Figure 9d and Figure 10b, the result shows that the effect of temperature on the Erichsen formability of woven CF/PEEK composite sheet is greater than the effect of the deformation speed on the Erichsen formability. When the deformation temperature is 325 °C and the deformation speed is 2 mm/min, the composite sheet with a rubbery-state matrix has the best Erichsen formability.

**Figure 11:**
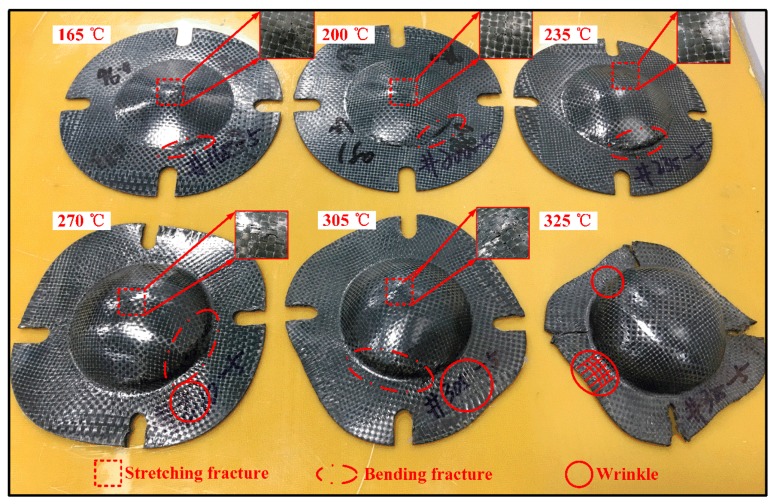
The fracture specimens of the Erichsen test of woven CF/PEEK sheets at different temperatures (5 mm/min).

**Figure 12:**
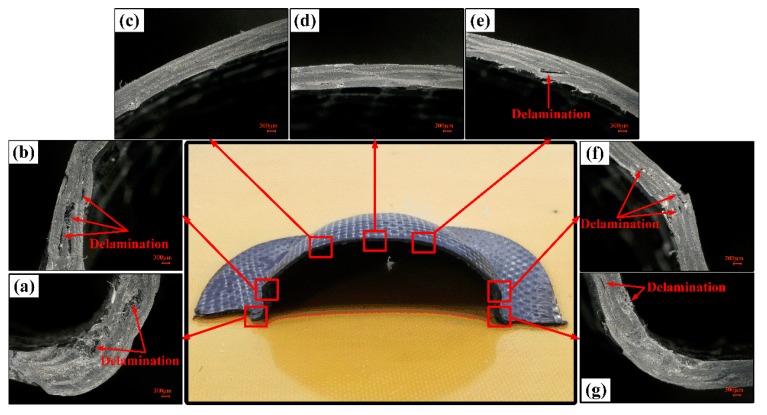
Cross-section of the fracture specimen of the Erichsen test under the conditions of 325 °C (IE = 32 mm).

Figure 11 shows the fracture specimens of woven CF/PEEK composite sheets at different deformation temperatures. As shown in Figure 11, the Erichsen formability of woven CF/PEEK sheets gradually increases with the increase of deformation temperature. When the deformation temperature is in the range of 165–235 °C, the failure of the woven CF/PEEK composite sheets are mainly caused by the stretching damage on the top and the bending damage at the shoulder. When the temperature rises to 270–305 °C, the failure of the sheets are caused by the stretching damage at the top, the bending damage at the shoulder, and the wrinkle damage at the flange. Especially, the bending damage at the shoulder gradually becomes the main failure mode, the crack expands from the compressed wrinkle at the upper surface to the fiber breaks on the lower surface as the deformation expands, which ultimately leads to the destruction of the shoulder. When the temperature rises to 325 °C, the failure of the sheet is mainly due to the delamination damage caused by wrinkles at the neck, shoulders and flanges, without the stretching and bending damage, which has shown in Figure 8 and Figure 12.

**Figure 13:**
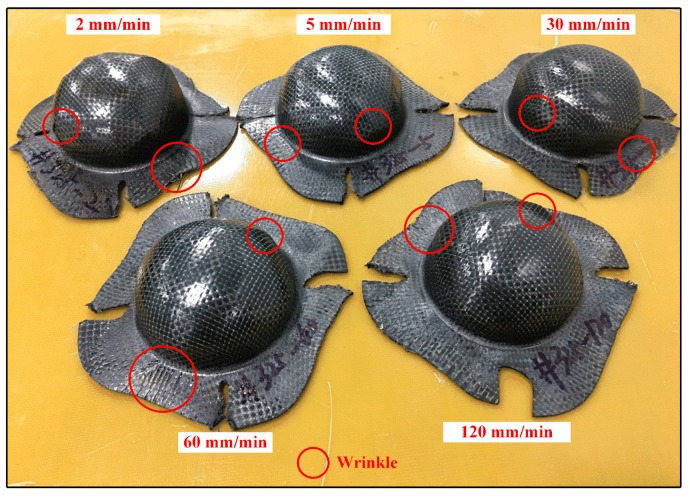
The fracture specimens of the Erichsen test of woven CF/PEEK sheets at different speeds (325 °C).

Figure 13 shows the fracture specimens of woven CF/PEEK composite sheets at different deformation speeds. As shown in Figure 13, the failure of sheets is due the delamination damage caused by wrinkles in the neck, shoulder, and flange positions when the deformation speed rises from 2 to 120 mm/min. Additionally, the wrinkle damage is larger when the deformation speeds are 2 mm/min and 120 mm/min, and it will be smaller when the deformation speed is in the range of 5–60 mm/min. In particular, the wrinkle of the woven CF/PEEK sheets during the Erichsen forming process mainly occurs at the position with [0°/90°] fibers, and few occur in the position with [−45°/45°] fibers, as shown in Figure 11 and Figure 13.

## 4. Discussions

**Figure 14:**
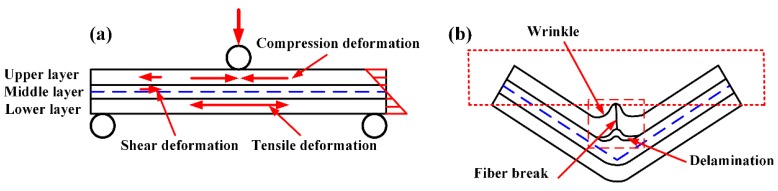
Schematic of flexural failure mechanism of woven CF/PEEK composites: (**a**) force schematic, and (**b**) failure schematic.

Figure 14 shows the schematic of flexural failure mechanism of woven CF/PEEK composites. In the flexural forming, three deformation states appear inside the specimens: the compression deformation of the upper layer, the shear deformation between the layers and the tensile deformation of the lower layer, and the deformation will be greater as it is closer to the surface, as shown in Figure 14a. The wrinkles is caused by the compression deformation of the upper layer. Since the deformation and relative slippage in the wrinkle region are the largest, fiber breakage and interlayer delamination first occur in this position, as shown in Figure 14b.

The increase in ambient temperature softens the PEEK matrix [7,32], increasing the ductility of matrix while attenuating the interaction of fiber/matrix [30,31]. When the temperature is in the range of 165–235 °C, the matrix exhibits weak toughness and stronger interaction of the fiber/matrix (see Figure 9a,b). The deformation of the fiber is restricted by the matrix, resulting in the small deformation and fiber fracture (see Figure 8a,b). As the temperature rises to 305 °C, the matrix strength and fiber/matrix interaction partially reduces, while the matrix toughness weakens (see Figure 9c), resulting in greater deformation, which causes fiber fracture and interlayer delamination (see Figure 8c). When the temperature (325 °C) is close to the Tm, the matrix strength and the interaction of fiber/matrix are extremely small while the matrix possesses strong toughness (see Figure 9b), so the large deformation of fiber filaments and layers are allowed without fiber fracture. Small matrix strength and large relative slippage lead to interlayer delamination (see Figure 8d). Therefore, the flexural properties of the composite decrease while the flexural formability increase when the temperature rises from 165 to 325 °C. Moreover, the wrinkles mainly cause fiber fracture at 165–305 °C, and interlayer delamination at 325 °C.

The increase of the strain rate will strengthen the PEEK matrix in the small deformation at elevated temperature, and the effect is not significant in the large deformations, which was proved in previous studies [7]. Meanwhile, more regions will participate in the deformation as the strain rate increases, resulting in more damage [33,34]. When the temperature is 325 °C, the composite sheets mainly fail in the large deformation. Therefore, the increase of the deformation speed has less influence on the matrix performance (see Figure 9e,f) but promotes more damage (see Figure 8e,f), resulting in a decrease in flexural failure strength and the formability of the composite sheet.

**Figure 15:**
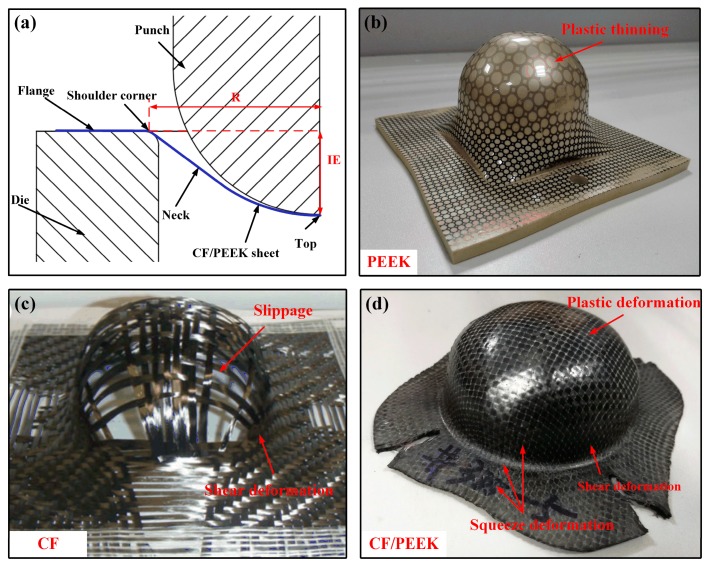
Erichsen test results of different materials at elevated temperature: (**a**) schematic of the Erichsen test, (**b**) PEEK tested, (**c**) CF, and (**d**) CF/PEEK.

Figure 15 is the Erichsen test results of different materials at elevated temperature. As shown in Figure 15a, the sheet is stretched at the top and bent at the shoulder in the small deformation stage. As the deformation increases, the stretched deformation gradually transfers to the neck, while the in-plane squeeze deformation occurs in the area near the shoulder. As shown in Figure 15b, the deformation of the pure PEEK is due to the plastic thinning of the top and neck in the Erichsen forming process, which was proved in previous studies [7]; while the deformation of the woven CF cloth is due to the slippage and shear deformation of the fibers [35], as shown in Figure 15c. However, in the solid-state thermoforming process, the deformation of the woven CF/PEEK composite sheet is mainly caused by plastic deformation, shear deformation, and squeeze deformation, and there is no plastic thinning and fiber slippage, as shown in Figure 15d. This is because the matrix is in the rubbery state and the fibers are locked by the interaction of fiber/matrix, the forming of the CF/PEEK sheets must overcome the deformation of the solid matrix and locked fibers.

When the deformation temperature is in the range of 165–235 °C, the deformation of the CF/PEEK composite sheets is small as the weak matrix toughness and strong fiber/resin interaction (see Figure 9a,b), resulting in the stretching and bending damages occur at the top and the shoulder, respectively. When the temperature is in the range of 165–235 °C, the matrix strength and fiber/matrix interaction partially reduces, while the matrix toughness weakens (see Figure 9c), resulting in greater deformation, which causes fiber fracture at the shoulder and wrinkling at the flange position. When the temperature rises to 325 °C, the matrix is close to Tm, the matrix has extremely low strength and strong toughness (see Figure 9d), and the restriction to the fiber deformation is also very weak, resulting in an excellent deformability of fiber and matrix. It is difficult to cause stretching damage at the top and bending damage at the shoulder even if the deformation is large. However, wrinkles caused by the in-plane squeeze deformation will occur at the neck, shoulders, and flange positions in the large deformation [3,35,36]. Therefore, the Erichsen formability increases when the temperature rises from 165 to 325 °C. Moreover, the failure of the sheets are primarily caused by fiber breakage at the top and shoulders at 165–305 °C, and it is due to interlayer delamination of the shoulders, neck, and flange at 325 °C. Since the temperature is 325 °C, a large Erichsen deformation of sheets occurs at the deformation speed of 2–120 mm/min. The increase in speed has less influence on the matrix performance (see Figure 9e,f) but promotes more damage (see Figure 8e,f), resulting in a decrease in Erichsen formability of the composite sheets.

**Figure 16:**
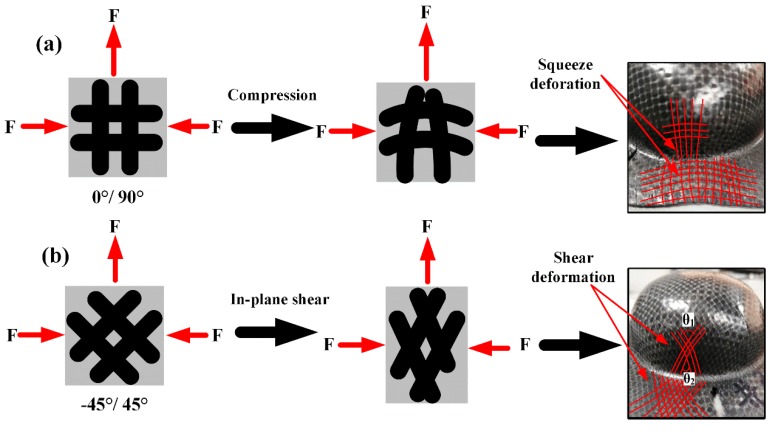
Schematic diagram of the deformation of woven unit in different fiber directions: (**a**) the [0°/90°] position, (**b**) the [−45°/45°] position.

Figure 16 is the schematic diagram of deformation of woven unit in different fiber directions. During the forming process, the shoulder area, the neck and flange area near the shoulder are subject to the pulling force and radial in-plane squeeze forces, which results in the in-plane compression deformation occurring in the [0°/90°] position (see Figure 16a) and in-plane shear deformation occurring in the [−45°/45°] position (see Figure 16b). In the [0°/90°] position, the wrinkles appear due to the squeeze of the matrix and fibers caused by the in-plane compression deformation while, in the [−45°/45°] position, the wrinkles will appear only when the shear angle θ1, θ2 caused by the in-plane shear deformation is greater than the “locking angle” [27,37].

## 5. Conclusions

In this paper, the formability of woven CF/PEEK composite sheets in the solid-state thermoforming process were investigated, and the failure mechanisms were systematically discussed. The conclusions are as follows:

(1) The flexural strength of woven CF/PEEK composite decreases as the deformation temperature and test speed are increased. However, the flexural modulus slightly decreases in the temperature range of 165–235 °C, and then significantly reduces as the temperature rises to 325 °C. Moreover, the flexural modulus tends to increase as the speed increases from 2 to 60 mm/min, and then decreases when the speed increase to 200 mm/min.

(2) The flexural and Erichsen test results show that it is feasible for thermoforming simple geometries of woven CF/PEEK sheets when the matrix is in a rubbery state (T_g_ < T < T_m_). The formability of CF/PEEK sheets significantly increases as the temperature rises from 165 to 325 °C, and slightly decreases as the deformation speed rises from 2 to 120 mm/min. The increase of temperature softens the matrix, increases the toughness of the matrix and reduces the restriction between matrix and fibers, which significantly increase the formability of the sheets. The increase of speed has less influence on the matrix performance in the temperature of 325 °C, but promotes more damage, which slightly decreases the formability of the sheets. When the temperature is 325 °C and the speed is 2 mm/min, the sheets possess good formability and forming quality.

(3) In the solid-state thermoforming process, the deformation of the woven CF/PEEK sheet is caused by plastic deformation, shear deformation, and squeeze deformation, and there is no plastic thinning and fiber slippage. This is because the matrix is in the rubbery state and the fibers are locked by the interaction of fiber/matrix, and the forming of the sheets must overcome the deformation of the solid matrix and locked fibers. Moreover, the wrinkles lead to fiber fracture at lower temperatures and delamination at higher temperatures. At higher temperatures, the wrinkles mainly occur at the position with [0°/90°] fibers due to the squeezing of the matrix and fibers, and few occur in the position with [−45°/45°] fibers.

## Figures and Tables

**Table 1:** Ericksen test conditions.

Experiment	Temperature/°C	Speed/mm/min	Blanking Torque/N/mm
1	165, 200, 235, 270, 305, 325	5	10
2	325	2, 5, 30, 60,120	10

## References

[1] Hernández S., Sket F., Molina-Aldareguı’a J.M., González C., Llorca J. (2011). Effect of curing cycle on void distribution and interlaminar shear strength in polymer-matrix composites. Compos. Sci. Technol..

[2] Schwab M., Todt M., Wolfahrt M., Pettermann H.E. (2016). Failure mechanism based modelling of impact on fabric reinforced composite laminates based on shell elements. Compos. Sci. Technol..

[3] Gong Y., Peng X., Yao Y., Guo Z. (2016). An anisotropic hyperelastic constitutive model for thermoplastic woven composite prepregs. Compos. Sci. Technol..

[4] Vaidya U., Chawla K. (2008). Processing of fibre reinforced thermoplastic composites. Int. Mater. Rev..

[5] Boudeau N., Liksonov D., Barriere T., Maslov L., Gelin J.C. (2012). Composite based on polyetheretherketone reinforced with carbon fibres, an alternative to conventional materials for femoral implant: Manufacturing process and resulting structural behaviour. Mater. Des..

[6] Dworak M., Rudawski A., Markowski J., Blazewicz S. (2017). Dynamic mechanical properties of carbon fibre-reinforced PEEK composites in simulated body-fluid. Compos. Struct..

[7] Zheng B., Wang H., Huang Z., Zhang Y., Zhou H., Li D. (2017). Experimental investigation and constitutive modeling of the deformation behavior of Poly-Ether-Ether-Ketone at elevated temperatures. Polym. Test..

[8] Tatsuno D., Yoneyama T., Kawamoto K., Okamoto M. (2017). Effect of Cooling Rate on the Mechanical Strength of Carbon Fiber-Reinforced Thermoplastic Sheets in Press Forming. J. Mater. Eng. Perform..

[9] Xu Z., Zhang M., Gao S., Wang G., Zhang S., Luan J. (2019). Study on mechanical properties of unidirectional continuous carbon fiber-reinforced PEEK composites fabricated by the wrapped yarn method. Polym. Compos..

[10] Xu Z., Zhang M., Wang G., Luan J. (2018). Bending property and fracture behavior of continuous glass fiber-reinforced PEEK composites fabricated by the wrapped yarn method. High Perform. Polym..

[11] Zheng B., Li M., Deng T., Zhou H., Huang Z., Zhou H., Li D. (2019). Process-structure-property relationships of thermoformed woven carbon-fiber-reinforced polyether-ether-ketone composites. Polym. Compos..

[12] Throne J.L. (2002). Thermoforming. Encyclopedia of Polymer Science and Technology.

[13] Gruenwald G. (2017). Thermoforming: A Plastics Processing Guide.

[14] Jiang W., Huang Z., Wang Y., Zheng B., Zhou H. (2018). Voids formation and their effects on mechanical properties in thermoformed carbon fiber fabric-reinforced composites. Polym. Compos..

[15] Novo P.J., Silva J.F., Nunes J.P., Marques A.T. (2016). Pultrusion of fibre reinforced thermoplastic pre-impregnated materials. Compos. Part B Eng..

[16] Schmitt R., Witte A. (2012). Control of a thermoplastic tape winding process with optical in-line metrology. Proc. Manuf. Syst..

[17] Bigg D. (1988). Mechanical property enhancement of semicrystalline polymers—A review. Polym. Eng. Sci..

[18] Bigg D., Preston J. (1989). Stamping of thermoplastic matrix composites. Polym. Compos..

[19] Cabrera N.O., Reynolds C.T., Alcock B., Peijs T. (2008). Non-isothermal stamp forming of continuous tape reinforced all-polypropylene composite sheet. Compos. Part A Appl. Sci. Manuf..

[20] Yanagimoto J., Ikeuchi K. (2012). Sheet forming process of carbon fiber reinforced plastics for lightweight parts. CIRP Ann..

[21] Uriya Y., Ikeuch K., Yanagimoto J. (2014). Cold and Warm V-bending Test for Carbon-fiber-reinforced Plastic Sheet. Procedis Eng..

[22] Uriya Y., Ikeuchi K., Yanagimoto J. (2014). Enhanced formability of thin carbon fiber reinforced plastic sheets in cold/warm embossing with ductile dummy sheets of different thicknesses. Int. J. Mater. Form..

[23] Uriya Y., Yanagimoto J. (2015). Suitable structure of thermosetting CFRP sheet for cold/warm forming. Int. J. Mat. Form..

[24] Uriya Y., Yanagimoto J. (2017). Erichsen cupping test on thermosetting CFRP sheets. Int. J. Mater. Form..

[25] Davey S., Das R., Cantwell W.J., Kalyanasundaram S. (2013). Forming studies of carbon fibre composite sheets in dome forming processes. Compos. Struct..

[26] Takuda H., Enami T., Kubota K., Hatta N. (2000). The formability of a thin sheet of Mg–8.5 Li–1Zn alloy. J. Mater. Process. Technol..

[27] Zhang Q., Gao Q., Cai J. (2014). Experimental and simulation research on thermal stamping of carbon fiber composite sheet. Trans. Nonferrous Met. Soc. China.

[28] Vieille B., Albouy W., Taleb L. (2014). Investigations on stamping of C/PEEK laminates: Influence on meso-structure and macroscopic mechanical properties under severe environmental conditions. Compos. Part B Eng..

[29] Yang S., Taha-Tijerina J., Serrato-Diaz V., Hernandez K., Lozano K. (2007). Dynamic mechanical and thermal analysis of aligned vapor grown carbon nanofiber reinforced polyethylene. Compos. Part B Eng..

[30] Nikforooz M., Montesano J., Golzar M., Shokrieh M.M. (2018). Assessment of the thermomechanical performance of continuous glass fiber-reinforced thermoplastic laminates. Polym. Test..

[31] Zheng B., Deng T., Li M., Huang Z., Zhou H., Li D. (2019). Flexural Behavior and Fracture Mechanisms of Short Carbon Fiber Reinforced Polyether-Ether-Ketone Composites at Various Ambient Temperatures. Polymers.

[32] Rae P.J., Brown E.N., Orler E.B. (2007). The mechanical properties of poly(ether-ether-ketone) (PEEK) with emphasis on the large compressive strain response. Polymer.

[33] Ou Y., Zhu D. (2015). Tensile behavior of glass fiber reinforced composite at different strain rates and temperatures. Constr. Build. Mater..

[34] Wang W., Zhang X., Chouw N., Li Z., Shi Y. (2018). Strain rate effect on the dynamic tensile behaviour of flax fibre reinforced polymer. Compos. Struct..

[35] Boisse P., Hamila N., Madeo A. (2016). Modelling the development of defects during composite reinforcements and prepreg forming. Philos. Trans. A Math. Phys. Eng. Sci..

[36] Alshahrani H., Hojjati M. (2017). Bending behavior of multilayered textile composite prepregs: Experiment and finite element modeling. Mater. Des..

[37] Gherissi A., Abbassi F., Ammar A., Zghal A. (2015). Numerical and Experimental Investigations on Deep Drawing of G1151 Carbon Fiber Woven Composites. Appl. Compos. Mater..

